# Histone methyltransferases regulate the transcriptional expression of ERα and the proliferation of tamoxifen-resistant breast cancer cells

**DOI:** 10.1007/s10549-019-05517-0

**Published:** 2020-01-02

**Authors:** Seung-Su Kim, Min-Ho Lee, Mi-Ock Lee

**Affiliations:** College of Pharmacy and Bio-MAX Institute, Research Institute of Pharmaceutical Sciences, Seoul National University, 1 Gwanak-ro, Gwanak-gu, Seoul, 08826 Korea

**Keywords:** Breast cancer, Tamoxifen resistance, MLL3, SET1A, ERα

## Abstract

**Purpose:**

Although tamoxifen remains the frontline treatment for ERα-positive breast cancers, resistance to this drug limits its clinical efficacy. Most tamoxifen-resistant patients retain ERα expression which may support growth and progression of breast cancers. Therefore, we investigated epigenetic regulation of ERα that may provide a rationale for targeting ERα in these patients.

**Methods:**

Expression levels of the mixed-lineage leukemia (MLL) family of proteins in tamoxifen-resistant breast cancer cells and publicly available breast cancer patient data sets were analyzed. Histone methylation levels in *ERα* promoter regions were assessed using chromatin immunoprecipitation. Expression levels of ERα and its target gene were analyzed using western blotting and real-time qPCR. Cell-cycle was analyzed by flow cytometry.

**Results:**

The expression of MLL3 and SET-domain-containing 1A (SET1A) were increased in tamoxifen-resistant breast cancers. An MLL3 chromatin immunoprecipitation-sequencing data analysis and chromatin immunoprecipitation experiments for MLL3 and SET1A suggested that these proteins bound to enhancer or intron regions of the *ESR1* gene and regulated histone H3K4 methylation status. Depletion of MLL3 or SET1A downregulated the expression level of ERα and inhibited the growth of tamoxifen-resistant breast cancer cells. Additional treatment with fulvestrant resulted in a synergistic reduction of ERα levels and the growth of the cells.

**Conclusions:**

The enhanced expression of MLL3 and SET1A in tamoxifen-resistant breast cancer cells supported the ERα-dependent growth of these cells by increasing ERα expression. Our results suggest that targeting these histone methyltransferases might provide an attractive strategy to overcome endocrine resistance.

**Electronic supplementary material:**

The online version of this article (10.1007/s10549-019-05517-0) contains supplementary material, which is available to authorized users.

## Introduction

Breast cancer is the most prevalent cancer in women and is one of the leading causes of cancer death in women worldwide [1]. Approximately 70% of breast cancers express the estrogen receptor (ER) and depend on ER signaling for their growth and progression [2]. By blocking estrogen binding to the ER and, thus, blocking the receptor’s action, tamoxifen has proven its efficacy and remains the frontline treatment for patients with ER-positive breast cancers, especially in premenopausal women [3]. Nonetheless, intrinsic and acquired resistance to tamoxifen limits its clinical efficacy. Almost half of patients with advanced disease do not respond to the drug and almost all patients with metastatic disease eventually develop resistance to it [3, 4]. Intriguingly, the majority of patients who have relapsed on tamoxifen treatment retain ERα expression [5, 6]. Several preclinical studies targeting ERα in tamoxifen-resistant cancers have yielded promising results [7–9]. Moreover, fulvestrant, a selective ER degrader, has shown clinical benefits in patients whose cancers have relapsed on tamoxifen, indicating that ERα might continue to play important roles in tamoxifen-resistant breast cancer growth [10–12]. Therefore, the delineation of novel mechanisms of regulation of transcriptional expression of ERα might provide therapeutic strategies to overcome tamoxifen resistance in ER-positive breast cancers.

Recent advances in genome-sequencing techniques have elucidated the wide distribution of epigenetic marks and mutations in DNA methyltransferases and histone-modifying enzymes, suggesting a direct link between alterations in the epigenome and cancer [13]. In particular, alterations in the levels of histone H3 lysine 4 (H3K4) methylation, which marks transcriptionally active sites, are generally associated with poor prognosis in renal, liver, and breast cancers [14–16]. Correspondingly, the mixed-lineage leukemia (MLL) family of proteins, MLL1, MLL2, MLL3, MLL4, SET-domain-containing 1A (SET1A), and SET1B, which all have the H3K4-methylating SET-domain, are frequently mutated in various cancers, including those of the breast, lung, large intestines, endometrium, and bladder [17]. In breast cancer tissues, comprehensive DNA sequencing revealed that the genes encoding MLL3 (*KMT2C*) and MLL4 (*KMT2D*) were two of the most frequently mutated cancer driver genes [18, 19]. SET1A is overexpressed in more than 10% of all breast cancer patients and modulates the proliferation and metastasis of breast cancer cells by regulating p53 target genes, such as *ARID3A*, *SESN1*, and *TP53INP1*, and by regulating a group of matrix metalloproteinases [20–22].

Several reports have demonstrated that the epigenetic control of estrogen-dependent transcription by the MLL-family of proteins promotes the progression of breast cancer [23–25]. Recent studies have highlighted the importance of these proteins by showing that MLL3 and MLL4 bind to the FOXA1 and ERα proteins in ER-positive breast cancer cells to control ER target gene transcription and proliferation [26, 27]. Furthermore, Manso et al. reported that genetic alterations leading to changes in MLL3 were enriched in tumor samples from patients with breast cancers who relapsed during adjuvant hormonal therapy [28]. Similarly, SET1A was demonstrated to be a factor that is required for the recruitment of TIP60 to ERα target gene promoter regions, and depletion of SET1A decreased estrogen-induced transcription [24]. However, the role of these proteins in tamoxifen resistance is largely unknown. Therefore, we aimed to test whether the proteins of MLL-family are involved in tamoxifen resistance in breast cancer cells.

## Methods

### Cell lines

The human breast cancer cell line MCF7 (HTB-22) and T47D (HTB-133) were obtained from the American Type Culture Collection (Manassas, VA, USA). MCF7 cells were grown in Dulbecco’s modified Eagle’s (DMEM) medium containing 10% fetal bovine serum (Hyclone, Logan, UT, USA) and T47D cells were grown in RPMI 1640 medium containing 10% fetal bovine serum. The tamoxifen-resistant and parent MCF7 sublines (MCF7/TAMR-1, MCF7/TAMR-8, and MCF7/S0.5) and T47D sublines (T47D/TR-1, T47D/TR-2, and T47D/S2) were obtained from Ximbio (London, UK). MCF7/S0.5 cells were maintained in phenol-red-free DMEM:F12 (1:1) containing 1% fetal bovine serum, 2 mM Glutamax^TM^ (Gibco-Invitrogen, Carlsbad, CA, USA), and 6 ng/ml of insulin (Merck KGaA, Darmstadt, Germany). T47D/S2 cells were maintained in phenol-red-free RPMI medium:F12 (1:1) containing 2% fetal bovine serum, 2 mM Glutamax^TM^, and 8 µg/ml of insulin. Tamoxifen-resistant cell lines were maintained in the media supplemented with 1 µM tamoxifen. Whenever 17β-estradiol (E_2_) was used, the cells were adapted to the media containing 2% charcoal-stripped FBS (Gibco-Invitrogen) for at least 48 h and the experiments were carried out in the same media. All cells were maintained under 5% CO_2_ in humidified air at 37 °C.

### Short interfering (si)RNA duplexes and transient transfection

siRNA duplexes targeting *MLL1*, *MLL2*, *MLL3*, *MLL4*, *SET1A*, *SET1B*, and the gene that encodes the nonspecific green fluorescent protein (GFP) were synthesized and purified by Bioneer Co. Ltd (Daejeon, Korea) (Supplementary Table 1). Transient transfection was performed as described previously [29].

### Western blot analysis, reverse transcription-quantitative PCR (RT-qPCR), and chromatin immunoprecipitation (ChIP)

Western blotting was carried out as described previously using specific antibodies against ERα, cyclin D1, c-Myc, histone H3 (Santa Cruz Biotechnology, Dallas, TX, USA), monomethylated histone H3K4 (H3K4me1), dimethylated histone H3K4 (H3K4me2), trimethylated histone H3K4 (H3K4me3) (Abcam, Cambridge, UK), and α-tubulin (Merck Millipore, Darmstadt, Germany). Band intensities of each protein were quantified using UVITEC (UVITEC, Cambridge, United Kingdom). RT-qPCR was carried out using specific primers, as described previously [29]. The sequences of the RT-qPCR primer used here are shown in Supplementary Table 1. ChIP assays were performed as described previously using specific antibodies against H3K4me1 and H3K4me3 (Abcam) [29]. The bound target DNA fragments (152,011,652 kb to 152,011,781 kb for ChIP1 and 152,128,814 kb to 152,128,940 kb for ChIP2) were detected using PCR. The primers used to amplify DNA fragments are given in the Supplementary Table 1.

### Cell proliferation and cell-cycle analysis

The proportion of viable cells was assessed on a hemocytometer using trypan blue exclusion. For cell-cycle assays, cells were harvested, fixed in 70% ethanol, stained with propidium iodide, and analyzed by flow cytometry (Becton Dickinson, Franklin Lakes, NJ, USA).

### Chromatin immunoprecipitation-sequencing (ChIP-seq) data analysis

MLL3 ChIP-seq data and their matched inputs (GSE81714) were downloaded from Gene Expression Omnibus (GEO; https://www.ncbi.nlm.nih.gov/geo/). Enriched regions of the genome were identified by comparing ChIP samples with input samples using the default parameters of the Model-based Analysis of ChIP-Seq peak-caller tool (v. 1.4.2) [30]. Peaks that were enriched by more than twofold over an input control with a *q*-value of < 0.05 were selected; 2876 peaks were identified in the MLL3 ChIP-seq data set. Genome-wide distributions were generated using the Integrative Genomics Viewer (https://software.broadinstitute.org/software/igv/) [31].

### Breast cancer patient cohort analysis based on public data sets

The publicly available data sets GSE9893, GSE3494, and GSE2034 were downloaded from GEO [32–34]. The GSE9893 data set (MLRG Human 21K v. 12.0; Montpellier Génopole Microarray core facility) contains the gene expression profiles of 155 patients treated with tamoxifen for 5 years after surgery. The GSE3494 data set (Affymetrix Human Genome U133A Array) contains the gene expression profiles of 315 patient cohort, among which 213 ER-positive breast cancer patient data was selected for analysis. The GSE2034 data set (Affymetrix Human Genome U133A Array) contains the gene expression profiles of 286 patient cohort, among which 209 ER-positive breast cancer patient data was selected for analysis. The RNA-seq data of The Cancer Genome Atlas (TCGA) breast invasive carcinoma cohort (https://cancergenome.nih.gov/, 601 ER-positive breast cancer patient samples), and the breast cancer patient gene expression profile data of the Netherlands Cancer Institute (NKI-295, 226 ER-positive breast cancer patient samples) were obtained from the Functional Genomics Explorer data bank (https://xena.ucsc.edu/; University of California, Santa Cruz, CA, USA) [35]. The Molecular Taxonomy of Breast Cancer International Consortium (METABRIC) and the GSE42568 data sets were obtained and analyzed in the CTGS website (https://ctgs.biohackers.net/) [36]. The METABRIC dataset contains gene expression profiles of 1980 patient cohort, of which 1398 ER-positive breast cancer patients were selected for analysis. The GSE42568 contains gene expression profiles of 121 patient cohort of which 67 ER-positive breast cancer patients were selected for analysis. No additional manipulation was performed on the data.

### Statistical analyses

Statistical analyses were performed using GraphPad Prism software (GraphPad Software, San Diego, CA, USA). Statistically significant differences between two groups were determined using two-tailed, paired Student’s *t* tests for protein quantification and unpaired Student’s *t* tests for any other analyses. Statistical analyses of multiple groups were performed using a two-way analysis of variance followed by a Bonferroni post-test correction. Data are reported as the mean ± SEM and *P* < 0.05 was considered significant.

## Results

### H3K4 methylation is increased in tamoxifen-resistant breast cancer

**Fig. 1:**
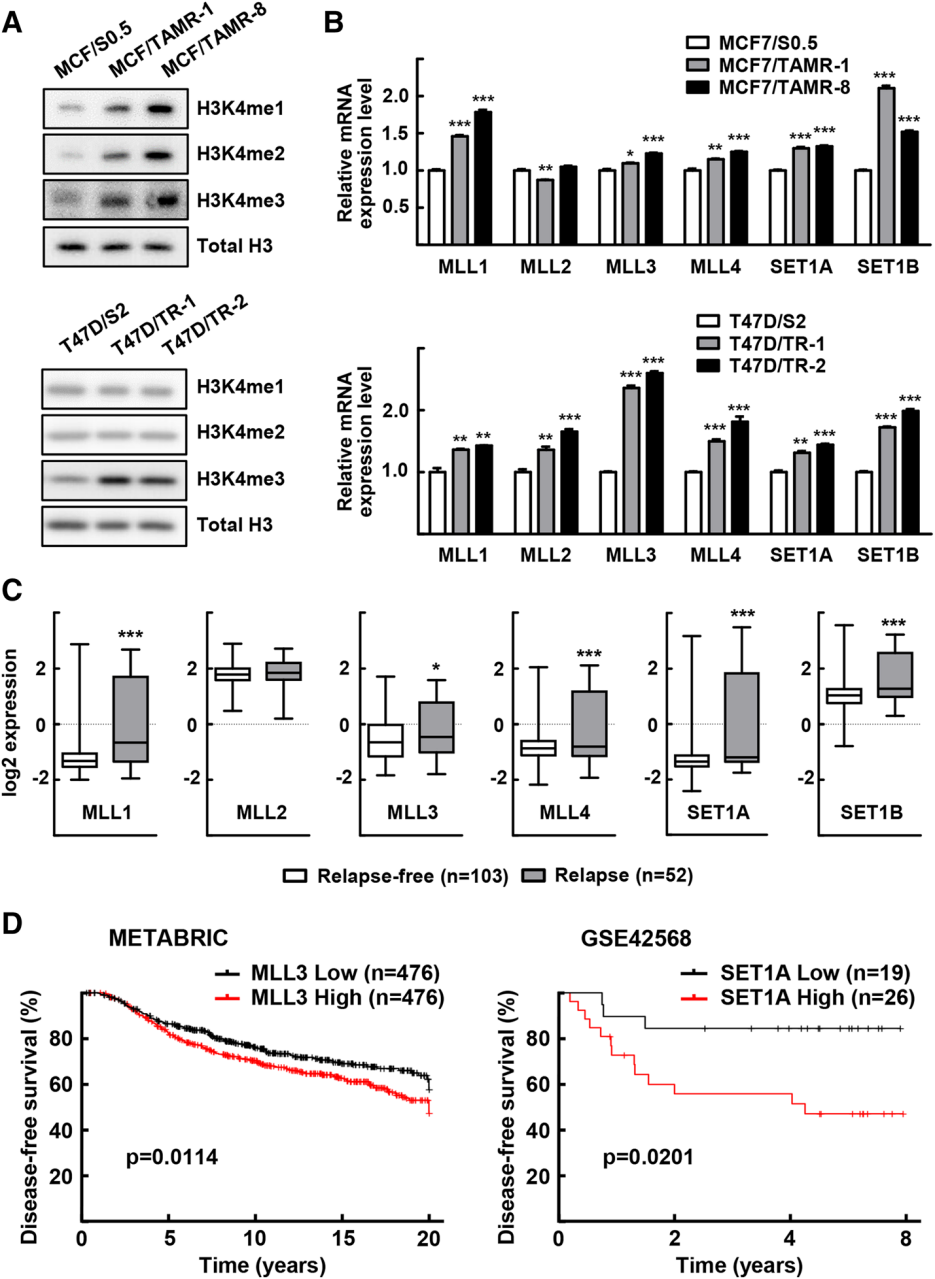
MLL expression is increased in tamoxifen-resistant breast cancer. **a**, **b** Total whole cell lysates and RNA obtained from the tamoxifen-resistant cells and their parental cells were subjected to western blotting (**a**) and qRT-PCR analysis (**b**). Data presented as mean ± SEM (*n* = 3). *P < 0.05, **P < 0.01 and ***P < 0.001. **c** The GSE9893 data set was obtained from NCBI GEO [32]. The expression levels of *MLL1, MLL2, MLL3, MLL4, SET1A,* and *SET1B* mRNA in 155 patients with ESR1-positive breast cancer treated with tamoxifen for 5 years after surgery are shown by the log2 expression value. *P < 0.05 and ***P < 0.001. **d** ER-positive breast cancer patient data from the METABRIC and the GSE42568 data sets were analyzed in the CTGS website (https://ctgs.biohackers.net/) [36]. Patients were categorized into a low gene expression (lower quartile) group and a high gene expression (upper quartile) group. Disease-free-survival rate (%) was plotted for each group. To analyze statistical differences, Log-rank (Mantel–Cox) tests were performed

To explore the role of epigenetic control in the development of tamoxifen resistance in breast cancer, H3K4 methylation levels were measured in the tamoxifen-resistant cells. We observed an increase in mono-, di-, and tri-methylation at H3K4 in the tamoxifen-resistant MCF-7 cells compared with parent cells, whereas H3K4me3 is enhanced in tamoxifen-resistant T47D cells (Fig. 1a and Supplementary Fig. 1). Because the proteins of the MLL-family are major enzymes that catalyze methylation at H3K4 residues, we analyzed the expression of these enzymes in the tamoxifen-resistant cells (Fig. 1b). Consistently, mRNA expression levels of most of the *MLL*-family genes were higher in the tamoxifen-resistant cells. To investigate the clinical relevance of this finding, we analyzed publicly available microarray data sets, which included gene expression profiles obtained from ER-positive breast cancer patients treated with tamoxifen. The expression levels of *MLL1*, *MLL3*, *MLL4*, *SET1A*, and *SET1B* were significantly higher in breast cancer tissues from the patients who relapsed compared with those who were relapse free (Fig. 1c) [32]. Moreover, the survival rates analyzed using the Kaplan–Meier method with the log-rank test in two independent public datasets, METABRIC and GSE42568, disease-free survival rate was significantly worse in the high MLL3 or high SET1A expression group (Fig. 1d). These results imply that these H3K4 methyltransferases might play a role in the development of tamoxifen resistance in breast cancer.

### MLL3 and SET1A regulate ERα gene expression

**Fig. 2:**
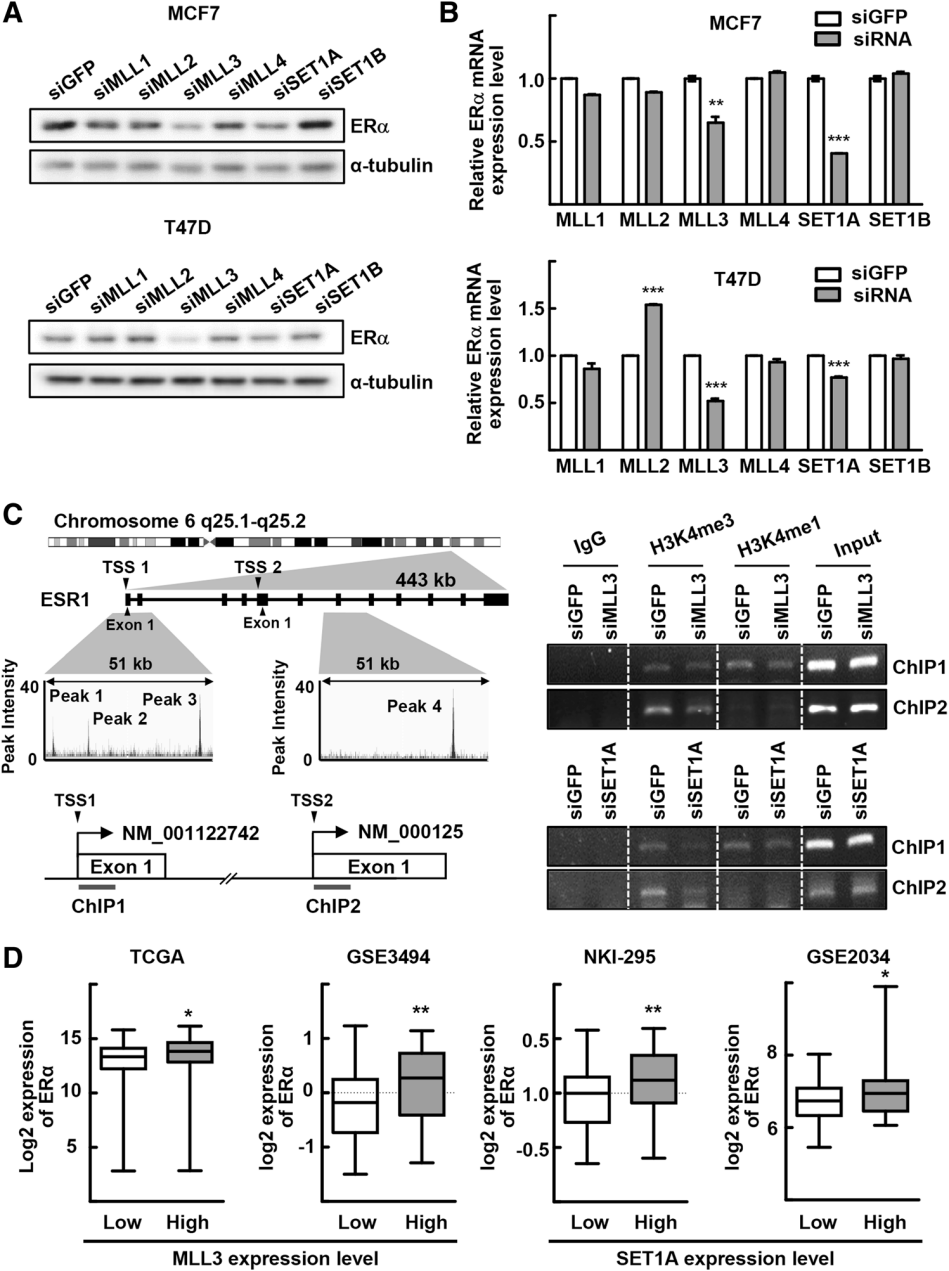
MLL3 and SET1A regulate ERα gene expression. **a**, **b** The MCF7 and T47D cells were transfected with indicated siRNA for 48 h. Total whole cell lysates and RNA obtained from MCF7 and T47D cells were subjected to western blotting (**a**) and qRT-PCR analysis (**b**), respectively. Data presented as mean ± SEM (*n* = 3). **P < 0.01 and ***P < 0.001. **c** Integrative Genomics Viewer tracks showed four MLL3 binding peaks in the ERα promoter/enhancer regions which was generated based on the analysis of the MLL3 ChIP-seq data set (GSE81714). Boxes and lines in the *ESR1* gene represent exons and introns, respectively (left top). Schematic representation of the human ERα promoter/enhancer regions for ChIP experiments (left bottom). MCF7 cells were transfected with siMLL3 or siSET1A for 48 h. DNA fragments that were immunoprecipitated by anti-H3K4me3 or anti-H3K4me1 antibodies were amplified by PCR using primers for ChIP1 and ChIP2 (right). TSS: transcriptional start site. **d** The mRNA expression of ERα and MLL3 in ER-positive breast cancer patients are based on TCGA RNA-seq or GSE3494 microarray data set (https://xena.ucsc.edu/) [33]. The mRNA expression of ERα and SET1A in ER-positive breast cancer patients are based on GSE2034 microarray data set or NKI microarray data set (https://xena.ucsc.edu/) [34, 35]. Patients were categorized into a low gene expression (lower quartile) group and a high gene expression (upper quartile) group. TCGA RNA-seq: *n* = 150 for MLL3-low and *n* = 150 for MLL3-High; GSE3494 microarray: *n* = 62 for MLL3-low and *n* = 62 for MLL3-High; NKI-295 microarray: *n* = 56 for SET1A-low and *n* = 56 for SET1A-High; GSE2034 microarray: *n* = 52 for SET1A-low and *n* = 52 for SET1A-High. *P < 0.05 and **P < 0.01

Tamoxifen-resistant breast cancer tissues and cell lines retain an elevated level of the ERα protein, which may drive cellular growth in these tamoxifen-resistant cells [6, 8, 9]. Therefore, we wondered whether genetic knockdown of the *MLL*-family genes changes *ERα* gene expression. First, we checked whether genetic knockdown of MLL-family genes affects ERα expression in breast cancer cells, MCF7 and T47D. Among the *MLL*-family genes, knockdown of MLL3 and SET1A resulted in a decrease in the ERα protein and mRNA levels, suggesting the transcriptional control of ERα expression by these histone-modifying enzymes (Fig. 2a, b and Supplementary Fig. 2a, b). The finding that alternative siRNA sequences targeting these genes also downregulated ERα mRNA expression significantly eliminated the possibility of potential off-target effects on ERα mRNA downregulation (Supplementary Fig. 2c).

Next, we explored whether MLL3 and SET1A control the expression of ERα by regulating *ERα* promoter activity directly in MCF7 cells. We identified the regulatory regions in the ERα-encoding genes as potential targets of MLL3 by analyzing recently reported MLL3 ChIP-seq data (Fig. 2c) [26]. Four MLL3 binding sites (peaks 1–4) were located at the enhancer or intron regions of the *ESR1* gene; peaks 1–3 were found near exon 1 of the ERα transcript NM_001122742 and peak 4 was located at an intron of the transcript NM_000125. Therefore, we examined whether the H3K4 methylation status in the *ESR1* gene was altered by MLL3. ChIP assays were performed using anti-MLL3 antibodies and two sets of PCR primers spanning the first exon of two different ERα transcripts, NM_001122742 and NM_000125. We found that knockdown of MLL3 reduced both H3K4me3 and H3K4me1 in the exon 1 region in both transcripts (ChIP 1 and ChIP 2), although H3K4me1 was not detected in the ChIP 2 region (Fig. 2c, Supplementary Fig. 2d). Interestingly, SET1A depletion yielded a similar pattern of histone methylation in the *ESR1* gene (Fig. 2c, Supplementary Fig. 2d). These results indicate that MLL3 and SET1A may regulate ERα expression by modifying the *ERα* promoter/enhancer regions via its histone methyltransferase activity. Consistent with these data, ERα mRNA levels in ER-positive breast cancer tissues were significantly higher in the cancer tissues with high expression of MLL3 or SET1A, as assessed based on the analysis of the publicly available breast cancer data sets (Fig. 2d) [33–35].

### Knockdown of MLL3 and SET1A decreases expression of ER-dependent growth-associated genes

**Fig. 3:**
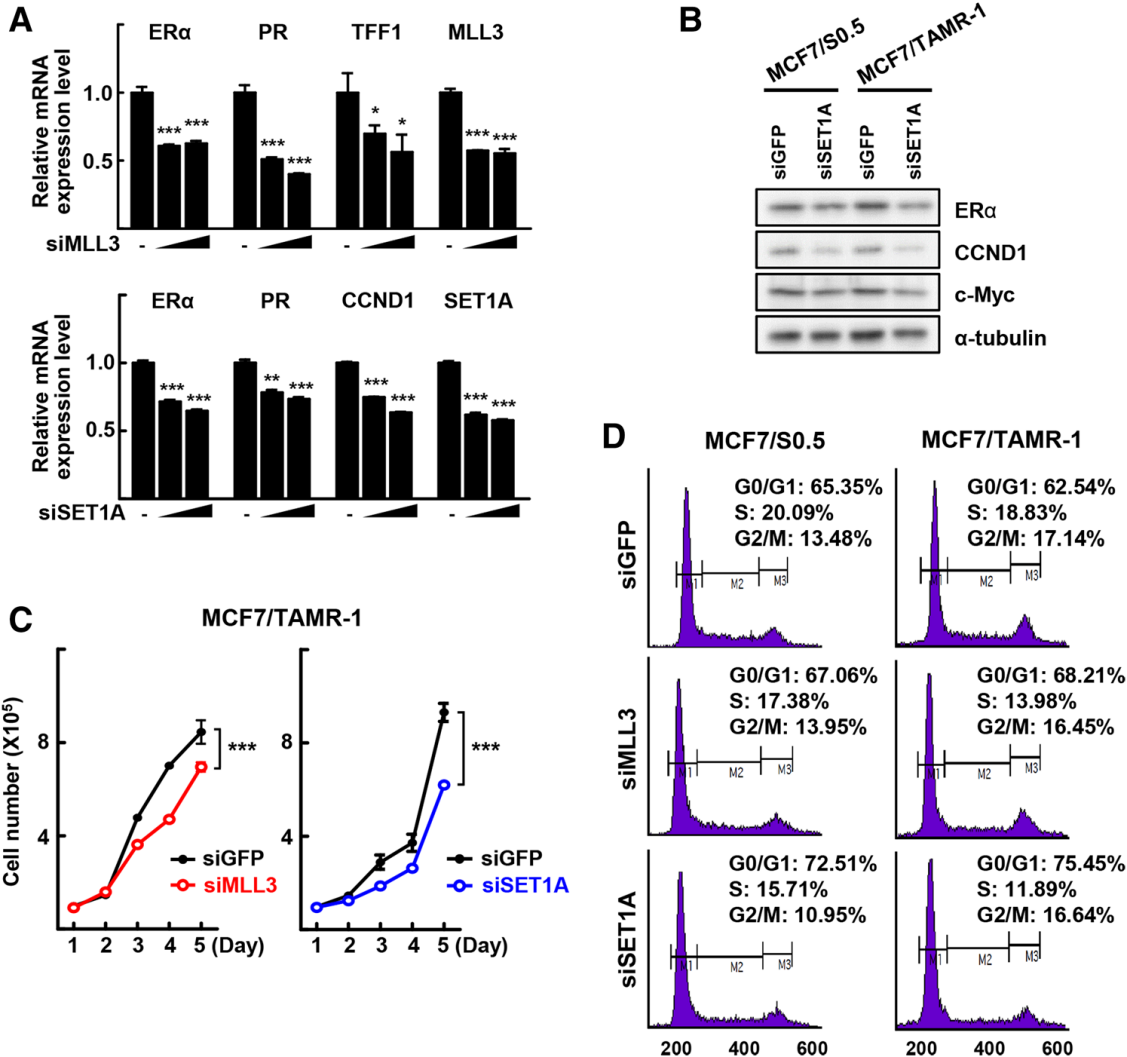
MLL3 and SET1A decrease gene expression of ERα and its downstream target genes and cell-growth. The MCF7, MCF7/S0.5, or MCF7/TAMR-1 cells were transfected with siMLL3 or siSET1A for 48 h. **a** Total RNA obtained from the MCF7 cells were subjected to qRT-PCR analysis. Data presented as mean ± SEM (*n* = 3). *P < 0.05, **P < 0.01 and ***P < 0.001. **b** The whole cell lysates obtained from MCF7/S0.5 or MCF7/TAMR-1 cells were subjected to western blotting. **c** The number of viable cells were counted using a hemocytometer. Cell numbers were presented as the mean ± SEM from duplicate plates. One of three independent experiments with similar results are presented. ***P < 0.001. **d** Transfected cells were stained with propidium iodide and analyzed by flow cytometry for cell-cycle status

Next, we examined whether knockdown of MLL3 or SET1A affected the expression of downstream targets of ERα, such as the progesterone receptor (*PR*), trefoil factor 1 (*TFF1*), and cyclin D1 (*CCND1*) genes. The mRNA levels of *ERα*, as well as those of *PR* and *TFF1*, were reduced significantly after knockdown of MLL3. Similarly, knockdown of SET1A decreased significantly the mRNA levels of *ERα*, *PR*, and *CCND1*, indicating that both MLL3 and SET1A are critical for the expression of ERα and its target genes (Fig. 3a). Similarly, knockdown of MLL3 and SET1A resulted in the reduced level of ERα and its target genes in both MCF7/S0.5 and MCF7/TAMR-1 cells (Supplementary Fig. 3a). Consistently, knockdown of SET1A reduced the levels of the ERα, CCND1, and c-MYC proteins (Fig. 3b). These downregulations were more obvious in the MCF7/TAMR-1 cells compared with their parent cells (Supplementary Fig. 3a, b). These results indicate that MLL3 and SET1A are responsible for the expression of ERα and its target genes, especially in tamoxifen-resistant breast cancer cells.

Several clinical trials support the notion that ERα is a pivotal driver of endocrine resistance in breast cancer and continues to be an important therapeutic target [5, 6]. Therefore, we investigated whether knockdown of MLL3 and SET1A inhibits the growth of the tamoxifen-resistant MCF7/TAMR-1 cells. Knockdown of MLL3 or SET1A decreased the number of MCF7/TAMR-1 cells by about 20% or 35%, respectively, after 5 days of culture (Fig. 3c). An E_2_ treatment increased largely the growth of MCF7/TAMR-1 cells, however the E_2_-induced growth was reduced significantly by knockdown of MLL3 or SET1A (Supplementary Fig. 3c). Although the E_2_ treatment increased the growth of parental cells, knockdown of MLL3 or SET1A affected marginally in the cell-growth regardless of the E_2_ treatment (Supplementary Fig. 3c). Cell-cycle analysis by flow cytometry showed that knockdown of MLL3 or SET1A increased the G0/G1 phase in MCF7/TAMR-1 cells by almost twofold compared with the control cells (Fig. 3d). These results demonstrated that the expression of ERα is important for the histone methyltransferase-associated inhibition of the growth of tamoxifen-resistant breast cancer cells.

### Inhibition of MLL3 and SET1A enhances fulvestrant sensitivity in tamoxifen-resistant cells

**Fig. 4:**
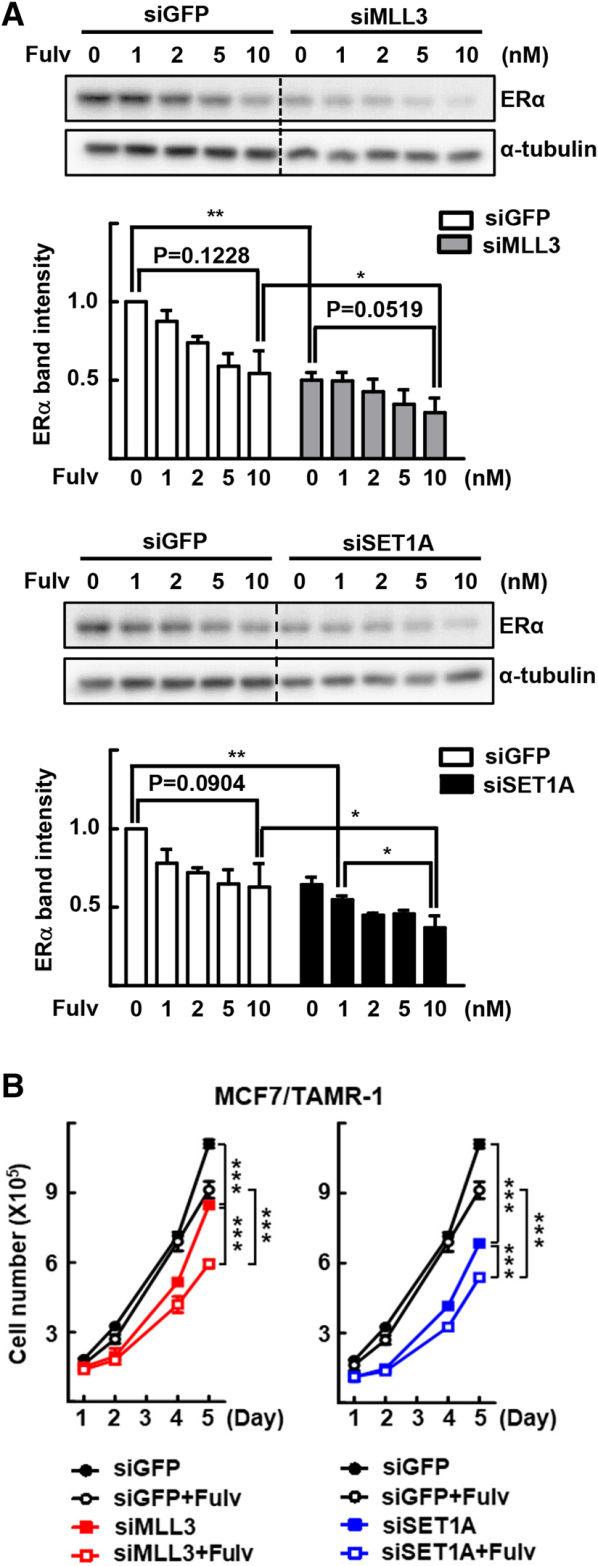
Inhibition of MLL3 and SET1A expression enhances sensitivity against fulvestrant-induced cell-growth inhibition in tamoxifen-resistant cells. **a** The MCF7/TAMR-1 cells were transfected with indicated siMLL3 or siSET1A for 48 h. Transfected cells were treated with the indicated concentration of fulvestrant (Fulv) for 24 h. The expression of ERα was determined by western blotting (top). Band intensities of ERα were quantified and normalized to that of α-tubulin. Data presented as mean ± SEM (*n* = 4). *P < 0.05 and **P < 0.01 (bottom). **b** The MCF7/TAMR-1 cells were transfected with indicated siMLL3 or siSET1A for 48 h. Transfected cells were treated with 5 nM fulvestrant for the indicated time periods and the number of viable cells was counted using a hemocytometer. Cell numbers were presented as the mean ± SEM from duplicate plates. One of three independent experiments with similar results are presented. ***P < 0.001

Finally, we evaluated the effect of fulvestrant, an ERα protein degrader, on the MLL3- or SET1A-knockdown-induced downregulation of ERα. We expected a further reduction of ERα levels based on two distinct mechanisms of ERα regulation, which may lead to enhanced sensitivity to fulvestrant of tamoxifen-resistant cells [10]. The ERα protein levels were decreased after treatment with fulvestrant in MCF7/TAMR-1 cells and were further decreased by knockdown of MLL3 or SET1A (Fig. 4a). Knockdown of MLL3 or SET1A together with fulvestrant treatment yielded an enhanced inhibition of the growth of MCF7/TAMR-1 cells, suggesting that ERα downregulation causes a reduction in cell-growth signaling (Fig. 4b).

## Discussion

In this study, we report for the first time that the H3K4 methyltransferases MLL3 and SET1A regulate the transcriptional expression of ERα by altering histone methylation patterns in the *ERα* promoter regions. In particular, the expression levels of MLL3 and SET1A were enhanced in the cancer tissues of patients with tamoxifen-resistant breast cancer, which may lead to the ERα-dependent proliferation of these cells (Figs. 1 and 2).

The histone methyltransferases of the MLL-family are highly conserved across eukaryotes and share the SET-domain, which is responsible for catalyzing histone methylation on H3K4 residues [37]. The MLL3 and SET1A members of this family may have redundancy on ERα transcription in breast cancer. For example, both MLL3 and SET1A modulate the histone methylation status near the MLL3 binding sites located in enhancer or intronic regions of the *ESR1* gene (Fig. 3). In particular, previous reports from our group and others have shown that the proximal region of the *ERα* promoter, including the exon 1 region in the NM_000125 sequence (ChIP2), is under the control of various epigenetic regulators, such as DNA methyltransferases and histone deacetylases [29, 38–40]. Furthermore, the ERα mRNAs transcribed from these two TSS sites are two of the four major ERα transcripts [41, 42]. The ERα transcript from NM_000125 represents half of the total ERα mRNA, and the ERα transcript from NM_001122742 represents almost 10% of the total ERα mRNA, which underscores the importance of the epigenetic function of both MLL3 and SET1A. Interestingly, a meta-analysis of 51 histone methyltransferases in 958 breast cancer samples revealed that SET1A is amplified, while MLL3 is mutated the most in the Luminal A subtype, which highlights the significance of MLL3 and SET1A in ERα signaling [43].

Our study, together with recent publications, highlights the important functions of MLL3 and SET1A in ERα signaling at two different levels. First, these histone methyltransferases regulate the transcriptional function of ERα by acting on ERα-binding sites. For example, Jozwik et al. showed that MLL3 forms a complex with ERα and FOXA1 in the enhancer regions of ERα target genes, such as *c-MYC* and *TFF1* [26]. Gala and colleagues also showed that MLL3 is necessary for ERα enhancer function, while its loss reprogrammed ERα to regulate its target genes via an AP-1-dependent mechanism, causing hormone independency [44]. Similar to the function of MLL3 on the transcriptional activity of ERα, SET1A facilitated the binding of ERα to the promoter/enhancer regions of ER target genes and increased estrogen-induced transcription [24, 45]. Accordingly, depletion of SET1A decreased the proliferation of ERα-positive and tamoxifen-resistant breast cancer cells [45]. Second, we showed here that MLL3 and SET1A upregulated ERα by inducing the active chromatin form on the promoter regions of the *ERα* gene (Fig. 2). Together, MLL3 and SET1A may act in a dual mode in ER-positive and tamoxifen-resistant breast cancer cells to confer ER-dependent breast cancer cell-growth, which could be therapeutically exploited by employing ERα antagonists, such as fulvestrant (Fig. 4).

Fulvestrant causes ERα protein destruction upon binding and has demonstrated clinical efficacy among patients who relapsed for a second time after responding to tamoxifen and aromatase inhibitors [10–12]. Fulvestrant treatment, as well as knockdown of the *ERα* gene, reduced the growth of tamoxifen-resistant breast cancer cells; moreover, this strategy was efficacious in several xenograft experiments [7, 46, 47]. In this study, we demonstrated that the combination of depletion of MLL3 or SET1A and fulvestrant treatment downregulated ERα and inhibited breast cancer cell-growth more effectively (Fig. 4). Therefore, this combination strategy may provide an insight into the regulation of the growth of tamoxifen-resistant breast cancer cells that have acquired alternative means of sustaining growth.

## Conclusions

Here, we report that the H3K4 methyltransferases MLL3 and SET1A regulate ERα expression epigenetically in ERα-positive breast cancer cells. We also found that MLL3 and SET1A were upregulated in tamoxifen-resistant breast cancer cells and that their depletion inhibited the expression of the target genes of ERα and repressed the proliferation of these cells. These effects were enhanced by treatment with fulvestrant. Thus, targeting these histone methyltransferases could provide an attractive strategy for overcoming endocrine resistance in patients with breast cancer.

## Electronic supplementary material

Below is the link to the electronic supplementary material.

Electronic supplementary material 1 (PDF 783 kb)

## Data Availability

All data generated or analyzed during this study are included in this published article and its supplementary information files. Any additional information is available upon reasonable request to the corresponding author.
