# Supplementary material for: Histone methyltransferases regulate the transcriptional expression of ERα and the proliferation of tamoxifen-resistant breast cancer cells
Source: Breast Cancer Res Treat. 2020 Jan 2;180(1):45–54. doi: 10.1007/s10549-019-05517-0 (PMC7031178; doi:10.1007/s10549-019-05517-0)
Supplement: Supplementary file 1 — Electronic supplementary material 1 (PDF 783 kb) [file 10549_2019_5517_MOESM1_ESM.pdf]

Supplementary Information

For *Breast Cancer Research and Treatment*

Histone methyltransferases regulate the transcriptional expression of ERα and the proliferation of tamoxifen-resistant breast cancer cells

Seung-Su Kim, Min-Ho Lee, and Mi-Ock Lee

College of Pharmacy and Bio-MAX Institute, Research Institute of Pharmaceutical Sciences, Seoul National University, 1 Gwanak-ro, Gwanak-gu, Seoul 08826, Korea

Corresponding Author: Mi-Ock Lee, College of Pharmacy, Seoul National University, 1 Gwanak-ro, Gwanak-gu, Seoul 08826, Korea. Phone: 82-2-880-9331; Fax: 82-2-887-2692; E-mail:molee@snu.ac.kr

Supplementary Table 1

Gene	Accession number		Nucleotide sequence	Species	Purpose	Reference
ERα	NM_000125<br>NM_001122742	Sense<br>Antisense	5'-CACATGAGTAACAAAGGCATGG-3'<br>5'-ATGAAGTAGAGCCCGCAGTG-3'	Human		[1]
MLL1	NM_001197104	Sense<br>Antisense	5'-GAAGTGGTTCCTGAGAATGG-3'<br>5'-CACAGTCGGAGAGATCATTTAG-3'	Human		-
MLL2	NM_014727	Sense<br>Antisense	5'-TGATCGAGAAAGTGCAAGAG-3'<br>5'-CTGGTGGTAACGGAACTTATAG-3'	Human		-
MLL3	NM_170606	Sense<br>Antisense	5'-AGTCTTCAGGAGGGTCTATG-3'<br>5'-CACAGGGAAGAGTGCTTTAG-3'	Human		-
MLL4	NM_003482	Sense<br>Antisense	5'-CTCTGGATGGGATTGATGCT-3'<br>5'-CGTGGCTCTTCCTGTTCTTC-3'	Human		-
SET1A	NM_014712	Sense<br>Antisense	5'-CATCGAATACGTGGGTCAGA-3'<br>5'-AATGCCCTCCTGCACGTA-3'	Human		[2]
SET1B	NM_001353345	Sense<br>Antisense	5'-GGGATCTGGACCAAAAACAA-3'<br>5'-CCCACGTAGAACTCATCGATCT-3'	Human		[2]
PR	NM_001202474	Sense<br>Antisense	5'-ACAGGACCCCTCCGACGAAAA-3'<br>5'-AGCTGTCTCCAACCTTGCACC-3'	Human	qRT-PCR primer	-
TFF1	NM_003225	Sense<br>Antisense	5'-ACCATGGAGAACAAGGTGAT-3'<br>5'-AAATTCACACTCCTCTTCTG-3'	Human		-
CCND1	NM_053056	Sense<br>Antisense	5'-GGATGCTGGAGGTCTGCGA-3'<br>5'-AGAGGCCACGAACATGCAAG-3'	Human		-
c-Myc	NM_002467	Sense<br>Antisense	5'-AAAGGCCCCCAAGGTAGTTA-3'<br>5'-GCACAAGAGTTCCGTAGCTG-3'	Human		
GREB1	NM_01466	Sense<br>Antisense	5'-GTGGTAGCCGAGTGGACAAT-3'<br>5'-ATTTGTTTCCAGCCCTCCTT-3'	Human		
β-actin	NM_001101	Sense<br>Antisense	5'-CGTGGGCCGCCCTAGGCACCA-3'<br>5'-TTGGCTTAGGGTTCAGGGGGG-3'	Human		-
18s rRNA	NR_145820	Sense<br>Antisense	5'-TGCATGGCCGTTCTTAGTTG-3'<br>5'-AGTTAGCATGCCAGAGTCTCGTT-3'	Human		-
ChIP 1	NM_001122742	Sense<br>Antisense	5'-CTACCGACTCAGAACGGATTT-3'<br>5'-CTCAGAGACTGTCTTCTTATGCT-3'	Human	ChIP primer	-
ChIP 2	NM_000125	Sense<br>Antisense	5'-AGGAGCTGGCGGAGGGCGTTCG-3'<br>5'-AGCGCATGTCCCGCCGACACGC-3'	Human		-
MLL1	NM_001197104	Sense	5'-AAGAAGUCAGAGUGCGAAGUC-3'	Human		-
MLL2	NM_014727	Sense	5'-GAAGGGCAUCGGGUGCUAUAU-3'	Human		-
MLL3	NM_170606	Sense	5'-GUGAUGAGGUGCGACAGAU-3'	Human		-
MLL3 #2	NM_170606	Sense	5'-GAGUGUUAGUGAGCCAUGA-3'	Human	siRNA	-
MLL4	NM_003482	Sense	5'-GCAGUUUGUGCACUCCAAG-3'	Human		-
SET1A	NM_014712	Sense	5'-GGAAAGAGCCAUCGGAAAUUU-3'	Human		-
SET1A #2	NM_014712	Sense	5'-GUCAGAGAACAGCUACCAA-3'	Human		-
SET1B	NM_001353345	Sense	5'-CAAGCUUGUUAUCAUGUAAAU-3'	Human		-

Supplementary Figures

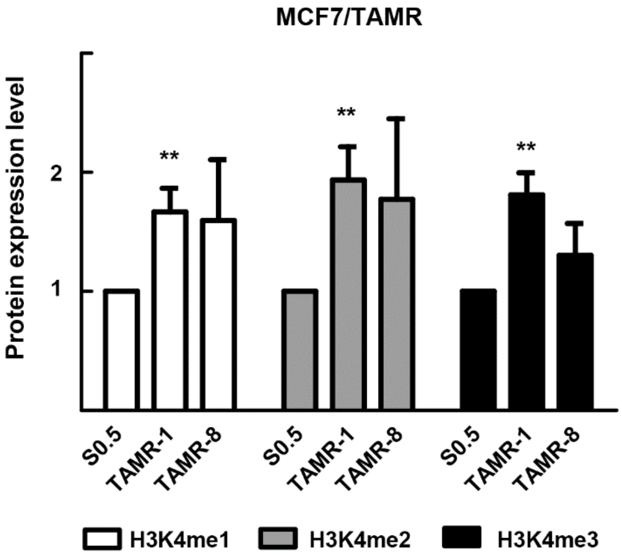

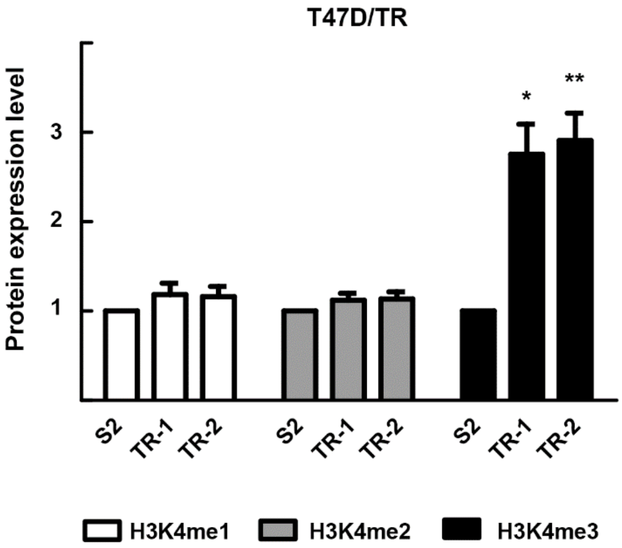

Figure 1. Histone H3K4 methylation status is increased in tamoxifen-resistant breast cancer cells. Band intensities of each protein in Figure 1A were quantified and normalized to that of Histone H3. Data presented as mean ± SEM (n=11 for MCF7 and n=4 for T47D). *, $P<0.05$ and **, $P<0.01$.

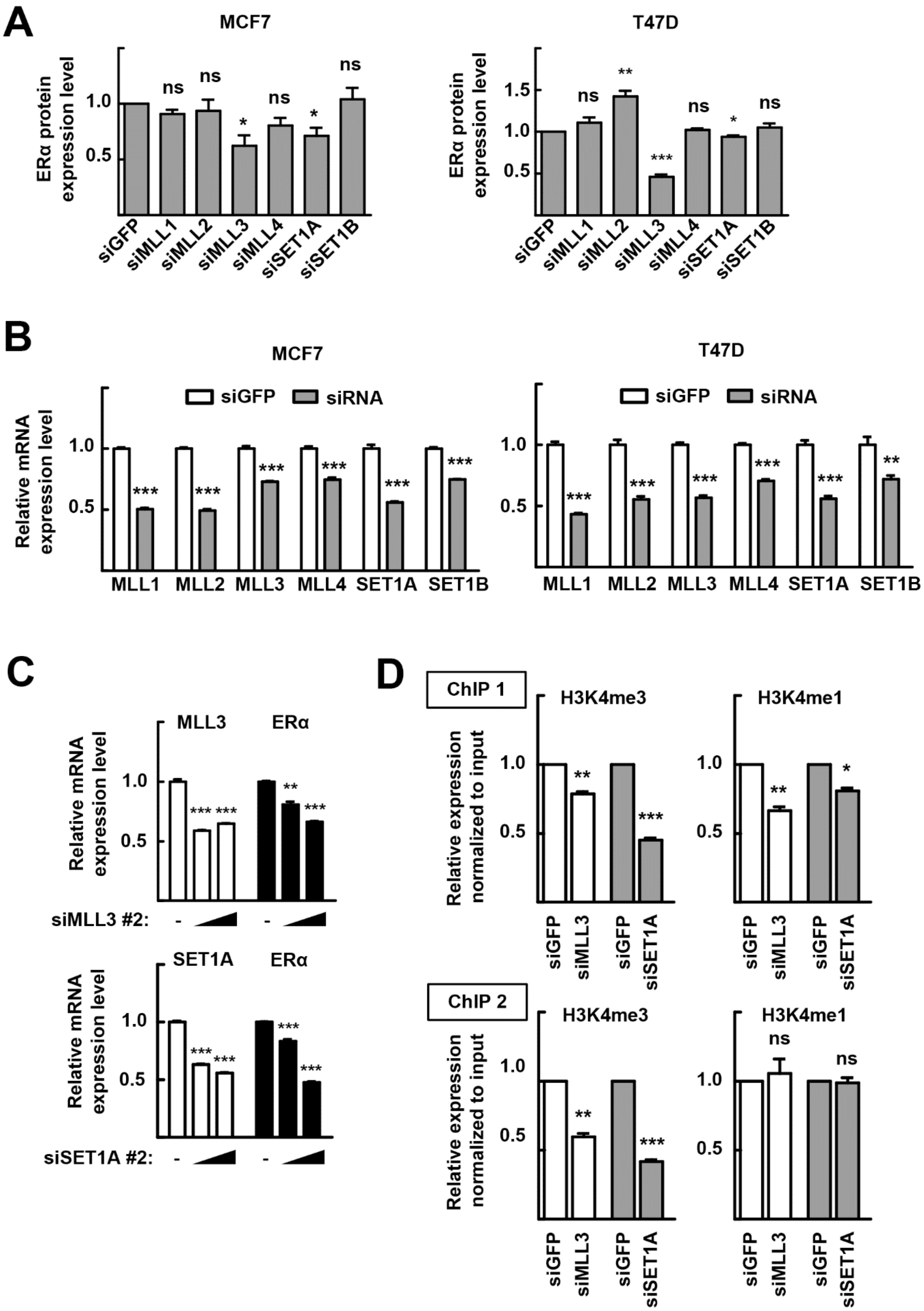
A
MCF7
T47D
ERα protein expression level
siGFP
siMLL1
siMLL2
siMLL3
siMLL4
siSET1A
siSET1B
B
MCF7
T47D
siGFP
siRNA
Relative mRNA expression level
MLL1
MLL2
MLL3
MLL4
SET1A
SET1B
C
MLL3
ERα
SET1A
siMLL3 #2:
siSET1A #2:
D
ChIP 1
ChIP 2
H3K4me3
H3K4me1
Relative expression normalized to input

Figure 2. MLL3 and SET1A regulate ERα gene expression

(A) Band intensities of each protein in Figure 2A were quantified and normalized to that of α-tubulin. Data presented as mean ± SEM (n=4). *, $P< 0.05$, **, $P<0.01$ and ***, $P< 0.001$.

(B) MCF7 and T47D cells were transfected with indicated siRNA for 48 h. Total RNA obtained from the MCF7 cells were subjected to qRT-PCR analysis. Each siRNA specific for MLL1, MLL2, MLL3, MLL4, SET1A, and SET1B downregulates the expression of each gene with 40~50% efficiency. Data presented as mean ± SEM (n=3). ***, $P< 0.001$.

(C) MCF7 cells were transfected with indicated siRNA for 48 h. Total RNA obtained from the MCF7 cells were subjected to qRT-PCR analysis. Data presented as mean ± SEM (n=3). **, $P<0.01$ and ***, $P< 0.001$.

(D) Band intensities of each DNA gel band in Figure 3C were quantified using ImageJ and normalized to that of input. Data represent three readings of each band as mean ± SEM (n=3). *, $P< 0.05$, **, $P<0.01$ and ***, $P< 0.001$.

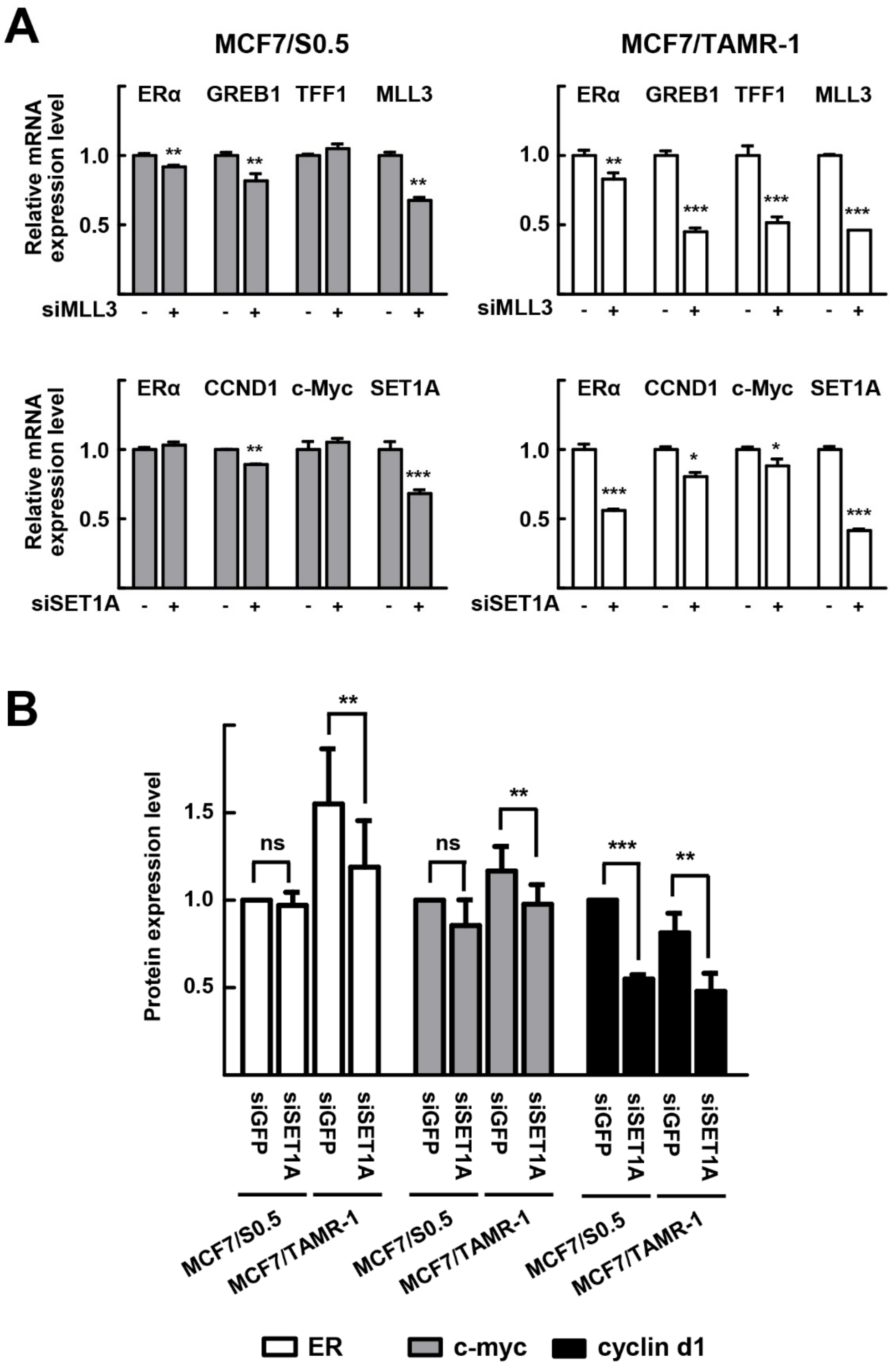
A
MCF7/S0.5
MCF7/TAMR-1
Relative mRNA expression level
ERα GREB1 TFF1 MLL3
siMLL3
ERα CCND1 c-Myc SET1A
siSET1A
B
Protein expression level
ns
**
***
siGFP
siSET1A
MCF7/S0.5
MCF7/TAMR-1
ER
c-myc
cyclin d1

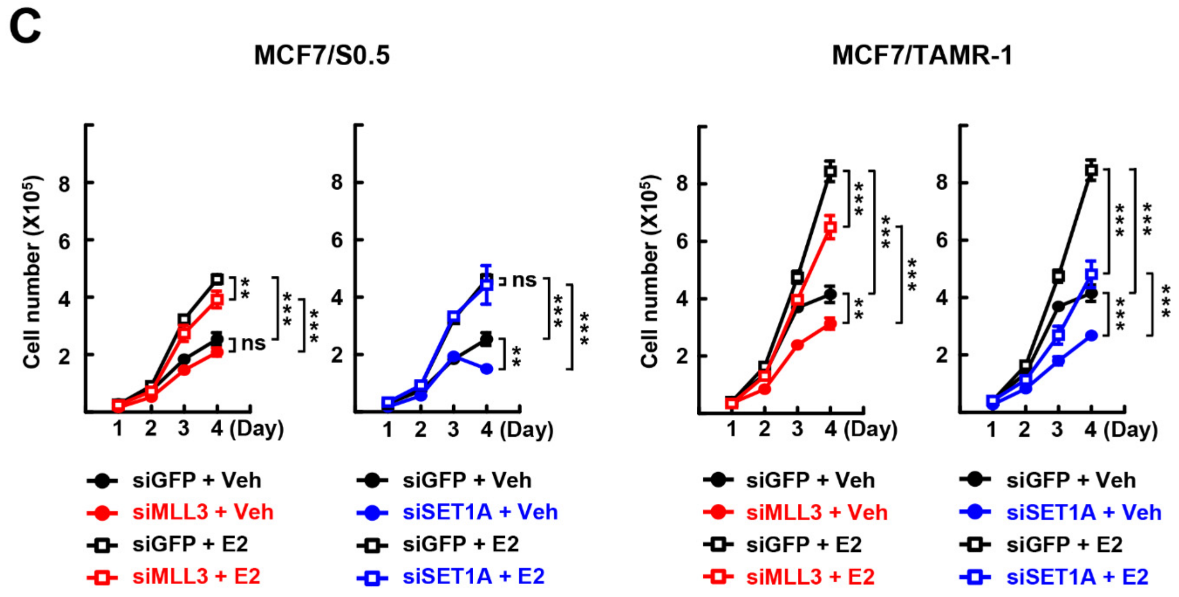

Figure 3. ERα target gene expression and cell growth inhibition was more obvious in the tamoxifen-resistant cells compared with their parent cells

(A) The MCF7/S0.5 and MCF7/TAMR-1 cells were grown in 2% charcoal-stripped FBS. The cells were transfected with siMLL3 or siSET1A for 48 h and 100nM E2 was treated for 24 h. Total RNA obtained from the MCF7/S0.5 and MCF7/TAMR-1 cells were subjected to qRT-PCR analysis. Data presented as mean ± SEM (n=3). *, P< 0.05, **, P< 0.01 and ***, P< 0.001.

(B) Band intensities of each protein in Figure 3B were quantified and normalized to that of α-tubulin. Data presented as mean ± SEM (n=6). *, P< 0.05, **, P<0.01 and ***, P< 0.001.

(C) The MCF7/S0.5 and MCF7/TAMR-1 cells were grown in 2% charcoal-stripped FBS. The cells were transfected with siMLL3 or siSET1A and 10nM E2 or vehicle was treated. The number of viable cells were counted using a hemocytometer. Cell numbers were presented as the mean±SEM from duplicate plates. One of two independent experiments with similar results are presented. **, P< 0.01 and ***, P< 0.001.

Supplementary Reference

1. Treeck O, Lattrich C, Springwald A et al (2010) Estrogen receptor beta exerts growth-inhibitory effects on human mammary epithelial cells. Breast Cancer Res Treat 120(3):557-65. doi: 10.1007/s10549-009-0413-2.

2. Castellini L, Moon EJ, Razorenova OV et al (2017) KDM4B/JMJD2B is a p53 target gene that modulates the amplitude of p53 response after DNA damage. Nucleic Acids Res 20;45(7):3674-3692. doi: 10.1093/nar/gkw1281.
